# Association between life’s essential 8 and periodontitis: a study based on NHANES 2009–2014

**DOI:** 10.3389/fmed.2024.1342792

**Published:** 2024-04-12

**Authors:** KeGui Hou, Hongli Zhang, Wenpeng Song, Shi Li, JiaRui Liu, Zhaofeng Ma

**Affiliations:** ^1^Beijing Shunyi District Hospital, Beijing, China; ^2^Beijing Tiantan Hospital, Capital Medical University, Beijing, China; ^3^Department of Stomatology, Seventh Medical Center of Chinese PLA General Hospital, Beijing, China; ^4^Shandong University of Traditional Chinese Medicine, Jinan, Shandong, China

**Keywords:** periodontitis, life’s essential 8, NHANES, epidemiology, risk factor(s)

## Abstract

**Background:**

This research aims to investigate the relationship between Life’s Essentials 8 (LE8), the American Heart Association’s latest indicator, and periodontitis. The purpose is to provide guidance on preventative measures.

**Methods:**

Data for our investigation were obtained from the National Health and Nutrition Examination Survey (NHANES) 2009–2014, with a total of 8,784 participants eligible. LE8 scores were compiled from 8 index scores (the score for each component of diet, physical activity, nicotine exposure, sleep duration, body mass index, blood lipids, blood glucose, and blood pressure). Periodontitis was classified by the Centers for Disease Control and Prevention and American Academy of Periodontology (CDC/AAP). The study utilized multivariable logistic analyses to investigate the potential correlation.

**Results:**

After controlling for all covariates, LE8 was discovered to have a significant negative correlation with periodontitis prevalence [0.91 (0.88, 0.94)]. This trend continued to hold statistical significance even after converting LE8 into a categorical variable. Furthermore, a noteworthy adverse correlation was discovered across both genders, specifically males [0.35 (0.22, 0.55)] and females [0.39 (0.25, 0.60)], as well as for the majority of categorical classifications, namely ethnicity, age, education level, and marital status. However, only the age subgroups displayed some degree of significant difference from each other.

**Conclusion:**

Life’s essential 8 was negatively associated with periodontitis, but more prospective trails are needed to confirm our findings.

## 1 Introduction

Periodontitis is a chronic inflammatory disease that damages the supporting tissue of the teeth, leading to the pathological resorption of the alveolar bone around the teeth, recession of the gingival tissues, and even loss of the teeth (1, 2). This condition poses a public health concern, impacting the oral and overall health of individuals across the world (3).

Matlila ‘s report represented the first reference to the relationship between oral infection and acute myocardial infarction (4). Subsequent research has shown that periodontitis not only affects systemic diseases but can also be influenced by them (5). Diseases such as cardiovascular disease trigger an immune response in the host, and the resulting metabolic dysfunction can cause chronic metabolic inflammatory disease. This, as one of the risk factors for periodontitis, can increase its morbidity (6, 7). Both periodontitis and cardiovascular disease are multifactorial conditions triggered by genetic, environmental, and lifestyle habits. Common risk factors for both diseases include increasing age, smoking, alcohol misuse, ethnicity, education and socioeconomic status, male gender, diabetes, and obesity. Several cross-sectional studies, case analyses, and epidemiological investigations indicate a significant correlation between chronic periodontitis and cardiovascular disease (8–11).

In 2010, the American Heart Association (AHA) defined “ideal cardiovascular health” as the presence of seven factors and behaviors that increase the chances of living a life free of cardiovascular disease and stroke (12). These seven factors and behaviors, including diet, physical activity, smoking, body mass index, total cholesterol, blood pressure, and blood glucose are known as “life’s simple 7 (LS7),” and are considered to be the core elements of building a healthier life (12). Studies have consistently demonstrated that higher LS7 scores are associated with greater cardiovascular health and reduced all-cause mortality in various populations (13–15). In 2022, the American Heart Association (AHA) established the Life’s Essential 8 (LE8) score, building upon the LS7 framework by introducing sleep as a novel cardiovascular health (CVH) determinant. According to the AHA, Life’s Essential 8 serves as a pivotal indicator for enhancing and preserving CVH, which can lower the incidence of heart disease, stroke, and other significant medical conditions (16–18). However, studies have not yet existed that have explored the relationship between LE8 and the incidence of periodontitis.

Hence, this study’s primary goal was to investigate this relationship, using nationally representative data from the National Health and Nutrition Examination Survey (NHANES).

## 2 Methods

### 2.1 Study population

**Figure 1:**
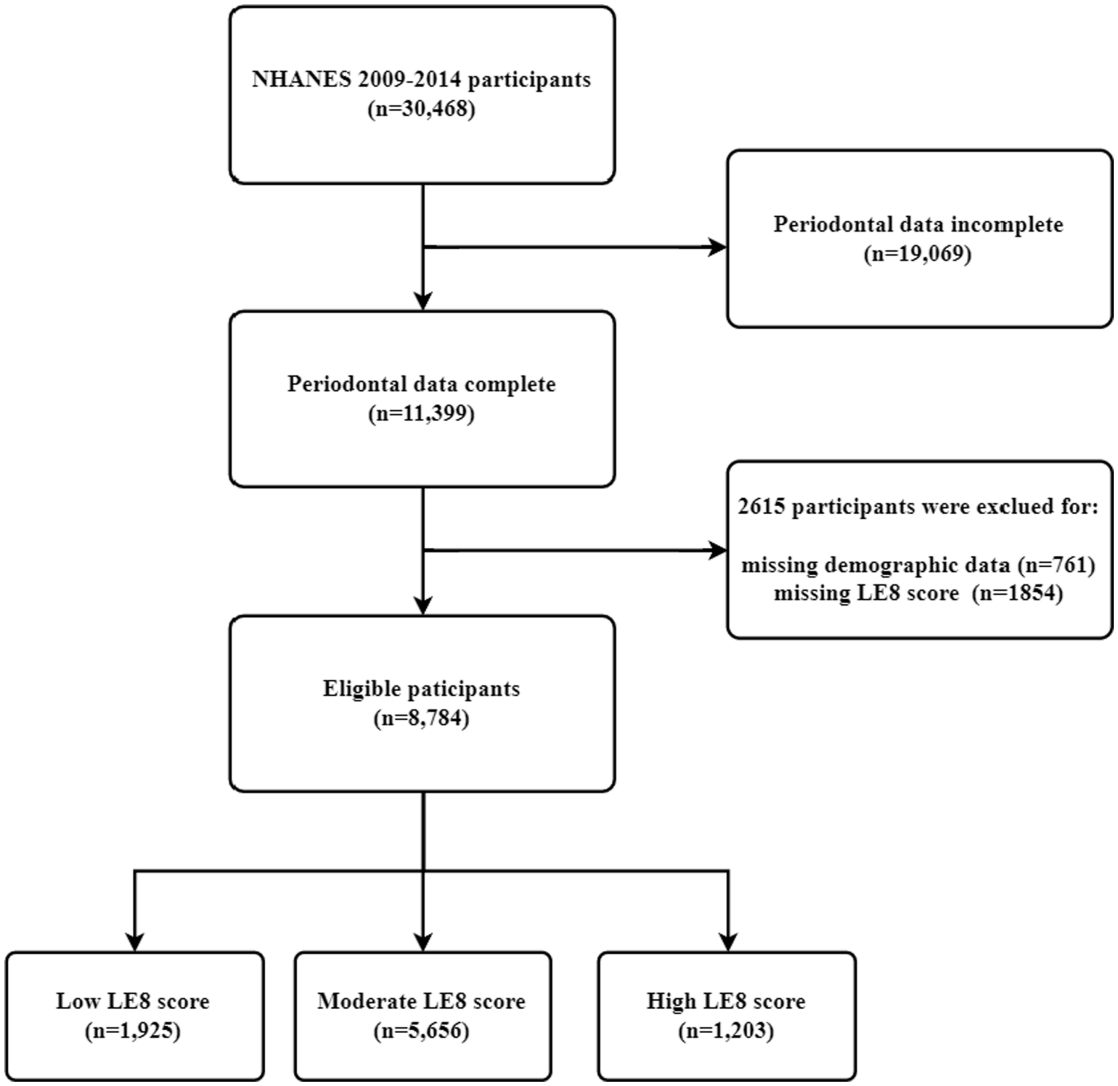
Study population selection (*N* = 8,784). NHANES, National Health and Nutrition Examination Survey; LE8, Life’s Essential 8.

The research data for this study were obtained from the NHANES survey from 2009–2014. NHANES is a population-based survey designed to collect health and nutritional information on the household population in the U.S.A (19). All participants provided written informed consent to conduct all survey procedures by relevant guidelines and standards.^1^ Over the 6 years of data from 2009–2014, as shown in Figure 1, a total of 8,784 respondents were finally included in the analysis of the study, with the following inclusion criteria: 18 years of age or older; having complete baseline information on the population; having received the NHANES “Oral Health-Periodontal Examination” and having recorded all measurements as required by the periodontal classification algorithm; and having the eight indicators as are necessary for a complete LE8.

### 2.2 Assessment of LE8

The LE8 metrics comprised four health behaviors and four health factors, namely diet, physical activity, nicotine exposure, sleep health, body mass index (BMI), non-high-density lipoprotein (HDL) cholesterol, blood glucose, and blood pressure (BP). These factors were evaluated as shown in Supplementary Table S1, where the HEI-2015 (The Healthy Eating Index-2015) was used to assess dietary levels on a scale from 0–100. Higher HEI scores indicated better diet quality (20). Day 1 total nutrient intake (DR1TOT) from NHANES was employed to compute the 13 elements of the HEI-2015 (21). Supplementary Table S2 provides specific guidance on how to calculate HEI-2015. The remaining 7 components can be derived directly from NHANES. LE8 scores were classified as low (0–49), moderate (50–79), or high (≥80) (15, 22).

### 2.3 Assessment of periodontitis

The program titled “Oral Health - Periodontal Screening” under the NHANES 2009–2014 performs measurements in six regions of every tooth with a maximum of 28 teeth. It contains two sets of clinical periodontal measurements - clinical attachment loss (CAL) and probing depth (PD). The periodontitis classification system was established based on case definitions from the Centers for Disease Control and Prevention and the American Academy of Periodontology (CDC/AAP). Severe periodontitis was determined by the presence of ≥2 interproximal areas with a CAL of ≥6 mm that were not on the same tooth and the presence of ≥1 interproximal area with a PD of ≥5 mm. Moderate periodontitis was defined as the presence of two or more interproximal sites with a probing pocket depth exceeding or equal to 5 mm, not located on the same tooth, or two or more interproximal sites with clinical attachment level exceeding or equal to 4 mm, not found on the same tooth, as previously established (23, 24). Moderate/severe periodontitis cases were identified as patients with periodontitis, while all other cases (no/mild periodontitis) were classified as the reference group (24, 25).

### 2.4 Treatment of covariates

Covariates included age (<40, 40–60, >60), gender (male, female), race (Mexican American, other Hispanic, non-Hispanic white, non-Hispanic black, different race/including multiracial), marital status (divorced/separated/married, married/cohabiting with a partner, never married), poverty-to-income ratio (categorized as low-income <1. 3, moderate-income 1.3–3.5, high income ≥3.5) (26), and educational attainment (less than high school, high school, some college or above).

### 2.5 Statistical analyze

All analyses were performed using R (version 4.2) and Empowerstats (version 5.0) (27, 28), and all statistical analyses were weighted according to the NHANES guidelines. Participants with high LE8 scores were considered to have LE8 scores of 80–100, moderate LE8 scores of 50 ~ 79, and low LE8 scores of 0 ~ 49 (16). We used chi-square tests and t-tests for LE8 trichotomous tests to assess demographic characteristics. To investigate the correlation between LE8 and periodontitis, we conducted a series of multiple linear regression analyses, examining the relationship between LE8 scores (per 10 points) and the prevalence of periodontitis. Finally, we conducted subgroup analyses to determine any differences in the above correlations based on gender, age, race, income, education, and marital status.

## 3 Results

### 3.1 Baseline characteristics

A study comprising 8,784 participants with an average age of 51.65 years was conducted, with 50.59% of the sample being male, as displayed in Table 1. The population prevalence of periodontitis was 49.44%. The LE8 scores were classified into low, medium, and high groups (0–49, 50–79, and 80–100) in agreement with the requirements prescribed by AHA. Overall, the prevalence of moderate to severe periodontitis was 49.44%. This decreased consistently with increasing LE8 group scores: 58.56% for the low group, 49.68% for the medium, and 33.25% for the high (*p* < 0.001). Moreover, higher LE8 scores were associated with a higher likelihood of being female, having higher education and income levels, and displaying significant differences in race and marital status.

**Table 1:** Participants’ Characteristics by LE8 Score.

LE8 score	Total (*n* = 8,784)	Low (*n* = 1,925)	Moderate (*n* = 5,656)	High (*n* = 1,203)	*p* value
Age (years)	51.65 ± 13.34	54.17 ± 12.59	51.89 ± 13.50	46.50 ± 12.35	<0.001
*Gender,* *n* (%)					<0.001
Male	4,444(50.59%)	1,005(52.21%)	2,952 (52.19%)	487 (40.48%)	
Female	4,340(49.41%)	920 (47.79%)	2,704 (47.81%)	716 (59.52%)	
*Race,* *n* (%)					<0.001
Mexican American	1,220(13.89%)	266(13.82%)	844 (14.92%)	110 (9.14%)	
Other Hispanic	841 (9.57%)	166 (8.62%)	574(10.15%)	101(8.40%)	
Non-Hispanic White	3,957(45.05%)	846(43.95%)	2,471(43.69%)	640 (53.20%)	
Non-Hispanic Black	1824(20.77%)	551(28.62%)	1,168(20.65%)	105(8.73%)	
Other Race/Including Multi-Racial	942 (10.72%)	96 (4.99%)	599(10.59%)	247(20.53%)	
*Marital status,* *n* (%)					<0.001
Divorced/Separated/Widowed	2,103(23.94%)	573(29.77%)	1,362(24.08%)	168 (13.96%)	
Married/Living with a partner	5,671(64.56%)	1,124(58.39%)	3,648(64.50%)	899(74.73%)	
Never married	1,010(11.50%)	228(11.84%)	646 (11.42%)	136 (11.31%)	
*Education level,* *n* (%)					<0.001
Less than high school	2062(23.47%)	689 (35.79%)	1,292 (22.84%)	81 (6.73%)	
High school	1941(22.10%)	542 (28.16%)	1,285 (22.72%)	114 (9.48%)	
Some college or above	4,781(54.43%)	694 (36.05%)	3,079 (54.44%)	1,008 (83.79%)	
Poverty-to-income ratio	2.61 ± 1.66	1.95 ± 1.44	2.62 ± 1.64	3.62 ± 1.56	<0.001
*Periodontitis (%)*					
Moderate /Severe	4,343(49.44%)	1,133 (58.86%)	2,810 (49.68%)	400 (33.25%)	
None/mild	4,441(50.56%)	792 (41.14%)	2,846 (50.32%)	803 (66.75%)	
*LE8 score*	62.23 ± 15.27	41.57 ± 6.46	64.08 ± 8.22	86.59 ± 5.06	<0.001
Health behaviors score					<0.001
HEI-2015 diet score	45.81 ± 32.38	26.16 ± 26.23	46.88 ± 31.13	72.24 ± 26.03	
Physical activity score	43.29 ± 46.59	9.88 ± 27.17	45.02 ± 46.40	88.65 ± 26.31	
Nicotine exposure score	69.52 ± 36.7	46.35 ± 38.95	72.71 ± 34.88	91.60 ± 18.12	
Sleep health score	79.89 ± 26.18	65.42 ± 30.84	82.28 ± 24.12	91.82 ± 15.55	
*Health factors score*					<0.001
Body mass index score	57.63 ± 33.55	36.12 ± 30.90	59.05 ± 31.77	85.32 ± 20.75	
Blood lipids score	60.96 ± 30.57	45.76 ± 30.04	62.03 ± 29.24	80.25 ± 24.74	
Blood glucose score	78.14 ± 27.44	59.61 ± 30.09	80.68 ± 25.26	95.86 ± 12.47	
Blood pressure score	62.57 ± 32.27	43.27 ± 30.21	63.94 ± 30.82	86.97 ± 21.78	

LE8: life’s essential 8; HEI: healthy eating index; Data were presented as weighted percentages or means (95% confidence intervals); A low score was defined as a LE8 score of 0 to 49, a moderate score of 50–79, and a high score of 80–100.

### 3.2 The association between LE8 and periodontitis

**Figure 2:**
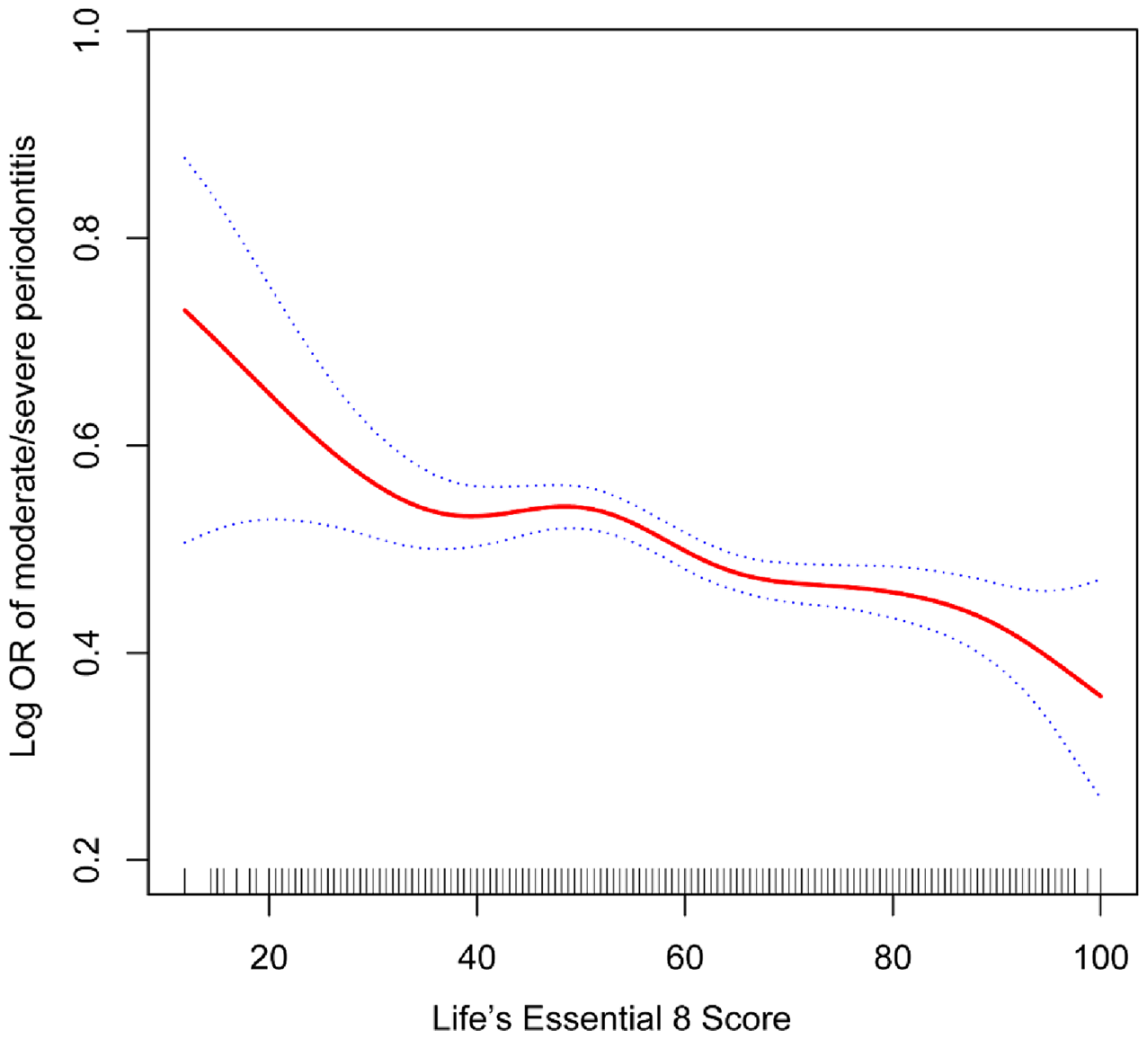
The association between life’s essential 8 and periodontitis. The solid red line represents the smooth curve fit between variables. Blue bands represent the 95% confidence interval from the fit.

The relationship between LE8 and periodontitis is presented in Table 2. It was found that LE8 scores had a negative association with the prevalence of periodontitis in all models. In the unadjusted model 1, the prevalence of periodontitis in the high LE8 group was 0.35 (95% confidence interval, 0.3, 0.4). In Model 3, after controlling for all covariates, including age, gender, race, income, marital status, and education level, the study revealed that the risk of periodontitis was reduced by 0.09% for every increase of 10 points in the LE8 score. In the high LE8 group, the risk decreases by 0.3%, and the results are statistically significant. Figure 2 illustrates the non-linear correlation between LE8 and periodontitis.

**Table 2:** Association between LE8 and periodontitis.

LE8	Model 1 OR(95%Cl) *p* value	Model 2 OR(95%Cl) *p* value	Model 3 OR(95%Cl) *p* value
Life’s Essential 8 (per 10 points)	0.79 (0.77, 0.82) <0.0001	0.84 (0.82, 0.87) <0.0001	0.91 (0.88, 0.94) <0.0001
*LE8 classification*			
Low (0–49)	Ref	Ref	Ref
Moderate (50–79)	0.69 (0.62, 0.77) <0.0001	0.72 (0.64, 0.80) <0.0001	0.84 (0.75, 0.94) 0.0018
High (80–100)	0.35 (0.30, 0.40) <0.0001	0.48 (0.41, 0.57) <0.0001	0.70 (0.59, 0.82) <0.0001
*P* for trend	<0.0001	<0.0001	<0.0001

Model 1 adjust for: None; Model 2 adjusts for Age; Gender, Race; Model 3 adjusts for Age, Gender, Race, PIR, Marital Status, and Education Level.

### 3.3 Subgroup analyses by potential effect modifiers

We conducted subgroup analyses of each covariate to determine potential effect factors. To illustrate the correlation between the two variables, we analyzed the association between LE8 score(per 10 points)and periodontitis. Table 3 presents results indicating age altering the association between LE8 scores and periodontitis. While several factors, including gender, race, income, education level, and marital status, had statistically significant effects within subgroups, they did not modify the correlation between LE8 and periodontitis between subgroups.

**Table 3:** Subgroup analyses by potential effect modifiers.

	LE8 (per 10 points)	P for interaction
*Gender*		0.7235
Male	0.35 (0.22, 0.55)	
Female	0.39 (0.25, 0.60)	
*Age*		0.0004
<40	0.88 (0.82, 0.95)	
40–60	0.86 (0.82, 0.90)	
>60	1.02 (0.97, 1.09)	
*Race*		0.7546
Mexican American	0.87 (0.79, 0.96)	
Other Hispanic	0.96 (0.86, 1.07)	
Non-Hispanic White	0.89 (0.85, 0.94)	
Non-Hispanic Black	0.89 (0.83, 0.95)	
Other Race/Including Multi-Racial	0.90 (0.81, 1.00)	
*Poverty-to-income ratio*		0.3296
<1.3	0.93 (0.87, 0.98)	
1.3–3.5	0.89 (0.84, 0.94)	
>3.5	0.89 (0.84, 0.94)	
*Education level*		0.2825
Less than high school	0.92 (0.86, 0.99)	
High school	0.92 (0.86, 0.98)	
Some college or above	0.88 (0.85, 0.92)	
*Marital status*		0.6032
Divorced/Separated/Widowed	0.90 (0.84, 0.96)	
Married/Living with a partner	0.91 (0.88, 0.95)	
Never married	0.89 (0.82, 0.98)	

^*^Each stratification was adjusted for age (continuous), sex (male, female), race (Mexican American, Other Hispanic, Non-Hispanic White, Non-Hispanic Black, Other Race/Including Multi-Racial), marital status (divorced/separated/widowed, married/living with a partner, and never married), poverty income ratio (classified as low income<1.3, middle income 1.3–3.5, and high income≥3.5), educational level (less than high school, 9–11th grade, high school, some college or above, ≥college graduate) except the stratification factor itself.

## 4 Discussion

In this cross-sectional study of large-scale, population-based survey data, we identified a negative association between LE8 scores and periodontitis. The negative association between LE8 scores and periodontitis was more significant in participants aged 40–60, according to subgroup analyses. As LE8 is a recent improvement for assessing cardiovascular health, the current report enhances the considerable evidence of an association between cardiovascular health and periodontitis (16). Improving LE8 scores may offer clinical benefits as a viable and effective means to promote periodontal well-being.

Cardiovascular health is a broader, more positive concept than simply the lack of illness. To measure cardiovascular health, the American Heart Association established LS7 and LE8 in 2010 and 2022 as tools for defining and quantifying cardiovascular health (16). The most recent LE8 employs a combination of two domains and eight metrics to determine one’s cardiovascular health. These metrics cover various health behaviors, including physical activity, diet, sleep health, and nicotine exposure, alongside health factors like blood pressure, blood glucose, blood lipids, and BMI.

A large number of studies have reported significant associations between most of the health factors and health behavior indicators in LS7 or LE8 and periodontitis individually, such as blood glucose (29), blood pressure (30), lipids (31), BMI (32), nicotine exposure (32, 33), and diet (34). However, to our knowledge, no study has evaluated the association between LS7 or LE8 as independent factors and periodontitis.

Healthy behaviors, such as a balanced diet and reduced nicotine exposure, can potentially prevent periodontitis. A cross-sectional study examining data from the US NHANES between 2009 and 2014 found that most identified dietary patterns were not correlated with periodontitis severity. However, a diet comprising mainly salads, fruits and vegetables, and plain water or tea was associated with reduced levels of CAL (34). Further data from the Hamburg City Health Study (HCHS), which examined 6,209 participants, indicated a noteworthy correlation between increased adherence to both the DASH diet and the Mediterranean diet and reduced likelihood of periodontal disease. It is essential to note that the Mediterranean Eating Pattern for Americans (MEPA) tool and the Healthy Eating Index scores-2015 (HEI-2015) in the LE8 scores can determine dietary healthiness. However, it is imperative to understand that these metrics do not assess the healthiness of all dietary patterns of individuals or populations (16). Nicotine exposure is described as using combustible tobacco, inhaling nicotine-delivery systems (NDS), or being exposed to secondhand smoke (16). A 2018 systematic review and meta-regression analysis reported an 85% increase in the risk of periodontitis due to cigarette smoking (35). Another study based on Mendelian randomization found a robust and reliable association between genetic predisposition to smoking and periodontitis (32). Exposure to environmental tobacco smoke has been positively linked to periodontitis endpoints, as demonstrated by prior research (33).

Interestingly, the risk of developing or progressing periodontitis in ex-smokers was not significantly different from that in never-smokers. The precise molecular mechanisms responsible for the negative correlation between smoking and periodontal tissue health are not fully understood (36). Still, current research suggests that it may be linked to smoking or nicotine exposure disrupting inflammation and the host’s response to periodontal pathogens, alterations to the subgingival microbial community, and hindered tissue healing potential (37).

Health factors such as good body mass index, lipids, blood glucose, and blood pressure contribute to maintaining periodontal health. In 2021, the results of a systematic total and meta-analysis based on a cohort study by Stöhr et al. (38) showed that there is a bi-directional positive correlation between periodontal disease and diabetes mellitus, i.e., diabetes mellitus increases the risk of periodontitis, and periodontal inflammation negatively affects blood glucose control. The association between diabetes and periodontal disease is underpinned by various factors, including hyperglycemia, genetic, microbial, and lifestyle co-predisposing factors, which culminate in advanced glycosylation end products (39). Additionally, poorly controlled diabetes can lead to elevated levels of IL1-β, TNF-α, IL-6, RANKL/OPG, and oxygen metabolites in the gingiva, which can contribute to the destruction of periodontal tissues (40). In addition, associations between obesity, hypertension, and dyslipidemia have also been reported with periodontal diseases (31, 41–44). On the other hand, improvement in systemic blood pressure and lipid levels is expected with periodontal treatment (30, 31).

The study discovered a link between LE8 (a marker of cardiovascular health) and periodontal health outcomes. This confirms previous research linking periodontitis and cardiovascular disease (45). Patients with periodontitis have an elevated risk of developing coronary heart disease and atherosclerosis (46–48). Effective periodontal care can significantly decrease the likelihood of experiencing cardiovascular events, including cardiac death and myocardial infarction, according to a report (49). The correlation between periodontitis and cardiovascular disease may be linked to bacteremia caused by the entry of periodontal pathogenic bacteria into the vascular system, an increase in the systemic inflammatory response due to periodontitis, genetic factors, and environmental risk factors (47).

Our research expands on the LE8, a clinical cardiovascular health assessment tool that is comprehensive and easy to use to indicate periodontal health outcomes. Adherence to the ideal Lifestyle Index 8 could be suitable for preventing and managing periodontal disease and cardiovascular health.

Our study has several strengths. First, NHANES utilizes a complex multistage probability sampling design that draws a representative noninstitutionalized resident population to ensure higher data quality. As a result, extrapolating the results to the entire U.S. civilian noninstitutional population is highly reliable. Second, we investigated for the first time the association between a new indicator, LE8, and periodontitis, which increases the feasibility of self-determination of periodontitis risk by people in their daily lives, increasing periodontitis prevention awareness and decreasing morbidity.

However, there are several limitations to the present study. Firstly, it is limited due to the cross-sectional nature of NHANES, which precludes the inference of causality and necessitates support from numerous future prospective studies. Secondly, data on health behaviors such as diet, exercise, sleep, and smoking are self-reported and may be prone to recall bias. Lastly, there are some unmeasured influences, and the experimental data may have errors.

## 5 Conclusion

In summary, the proposed LE8 index by the American Heart Association revealed a negative correlation with the risk of periodontitis. Furthermore, the association was stronger in participants aged between 40 and 60 years, and LE8 exhibited a nonlinear correlation with the incidence of periodontitis. These findings imply that improving the LE8 index could be an effective measure for the tertiary prevention of periodontitis.

## Data availability statement

The original contributions presented in the study are included in the article/Supplementary material, further inquiries can be directed to the corresponding author.

## Ethics statement

Ethical approval was not required for the study involving humans in accordance with the local legislation and institutional requirements. Written informed consent to participate in this study was not required from the participants or the participants' legal guardians/next of kin in accordance with the national legislation and the institutional requirements.

## Author contributions

KH: Writing – original draft. HZ: Data curation, Writing – review & editing. WS: Software, Writing – original draft. SL: Investigation, Writing – review & editing. JL: Methodology, Writing – original draft. ZM: Writing – review & editing.

## Footnotes

^1^ https://www.cdc.gov/nchs/data_access/restrictions.htm
